# Loss of an eye to mucormycosis following corticosteroid therapy for COVID-19

**DOI:** 10.4322/acr.2021.345

**Published:** 2021-09-12

**Authors:** Mariana Gonçalves Rodrigues, William Kazunori Sekiguchi, Sérgio Gonçalves, Yuri Reis Casal, Fernando Pereira Frassetto, Vinicius Trindade Gomes da Silva, Marcelo Prudente do Espírito Santo, Marcello Mihailenko Chaves Magri

**Affiliations:** 1 Universidade de São Paulo (USP), Hospital das Clínicas da Faculdade de Medicina, Disciplina de Cirurgia de Cabeça e Pescoço, São Paulo, SP, Brasil; 2 Universidade de São Paulo (USP), Hospital das Clínicas da Faculdade de Medicina, Departamento de Moléstias Infecciosas, São Paulo, SP, Brasil; 3 Universidade de São Paulo (USP), Hospital das Clínicas da Faculdade de Medicina, Departamento de Patologia, São Paulo, SP, Brasil; 4 Universidade de São Paulo (USP), Hospital das Clínicas da Faculdade de Medicina, Departamento de Neurocirurgia, São Paulo, SP, Brasil

**Keywords:** Keywords:, Immunocompetence, SARS-CoV-2, Orbit

## Abstract

Mucormycosis is a rare, sometimes severe fungal infection that has emerged as a possible complication of COVID-19. We report a case of a non-diabetic, apparently immunocompetent patient diagnosed with rhino-orbital-cerebral mucormycosis shortly after COVID-19 treatment with dexamethasone. The patient received optimized systemic antifungal therapy and extensive surgical treatment. So far, four months after the last hospital discharge, the patient has been in good general condition. This case is a dramatic reminder that beneficial corticosteroid therapy in general inevitably carries a risk of opportunistic infection, and corticosteroid therapy for COVID-19 risks orbital-rhinocerebral mucormycosis that clinicians should watch for with vigilance.

## INTRODUCTION

Mucormycosis is the infection caused by fungi of the phylum *Mucoromycota*, order *Mucorales*. *Mucorales* fungi are ubiquitous and rarely cause infection in healthy patients. The common underlying diseases in mucormycosis include diabetes mellitus, hematological malignancies, solid organ tumor, hematopoietic stem cell and solid organ transplants, corticosteroid therapy, and neutropenia.^1^

Mucormycosis’ diagnosis depends on a high index of suspicion due to its rarity outside endemic regions. In this setting, imaging tests and laboratory investigations are essential. Histopathologically, the hyphae are non or pauci-septated and appear ribbon-like with an irregular branching pattern.^1^

During the COVID-19 pandemic, systemic fungal infections are causing concern.^2^ Most patients reported with COVID-19-associated mucormycosis had diabetes, but there are other possible risk factors in the pandemic context, such as corticosteroids use during moderate and severe forms of COVID-19 treatment.^3^

We report a case of a previously healthy patient diagnosed with rhino-orbital-cerebral mucormycosis shortly after COVID-19 treatment.

## CASE REPORT

A 39-year-old female patient had flu-like symptoms and was diagnosed with COVID-19 by polymerase chain reaction of the oro/nasopharyngeal secretion. She had no diabetes mellitus history (HbA1c 5,5% in the admission) nor other illness. She was considered immunocompetent (Table 1 shows laboratory investigation towards the patient's immunological status).

**Table 1:** Immunologic laboratory workup data

	result	RV			result	RV
HbA1c	5.5%	4.7-5.6%		Proteins	7.6 g/dL	6-8 g/dL
Leukocytes	9300/mm^3^	4000-11000/mm^3^		albumin	5 g/dL	3.2-5.0 g/dL
Neutrophils	5520/mm^3^	2500-7500/mm^3^		Alfa-1 g	0.3 g/dL	0.1-0.3 g/dL
Lymphocytes	2430/mm^3^	1500-3500/mm^3^		Alfa-2 g	0.7 g/dL	0.6-1.0 g/dL
Monocytes	880/mm^3^	200-800/mm^3^		Beta-1 g	0.5 g/dL	0.4-0.7 g/dL
Basophils	100/mm^3^	0-100/mm^3^		Beta-2 g	0.3 g/dL	0.3-0.4 g/dL
Eosinophils	370/mm^3^	4-440/mm^3^		Gamma g	0.7 g/dL	0.8-1.6 g/dL
Platelets	199.10^3^/mm^3^	15010^3^-40010^3^/mm^3^		HIV	Non-reactive	Non-reactive

g = globulin; RV = reference value.

The patient evolved with dyspnea and hypoxemia, requiring hospitalization and oxygen supplementation. According to a local protocol for COVID-19 treatment, she received dexamethasone (8 mg/day for 5 days), ivermectin, rivaroxaban and azithromycin. Fifteen days later, she had severe pain, edema, and redness over the left eye. Within hours, this condition evolved to a transitory loss of consciousness episode and sphincter release, which motivated a new hospitalization. At hospital admission, the patient had left amaurosis. Periorbital cellulitis and rhinosinusitis (left maxillary, ethmoid and sphenoid sinuses) were identified by imaging exams. She underwent endoscopic endonasal sinusectomy five days later, and received piperacillin/tazobactam and amikacin for eleven days. Fifteen days after hospital discharge, she presented spontaneous drainage of a left upper eyelid abscess, treated with clindamycin. Two weeks later, she underwent a new sinusectomy, which still found necrotic tissue. The patient received ciprofloxacin, vancomycin, and amphotericin for 7 days.

**Figure 1:**
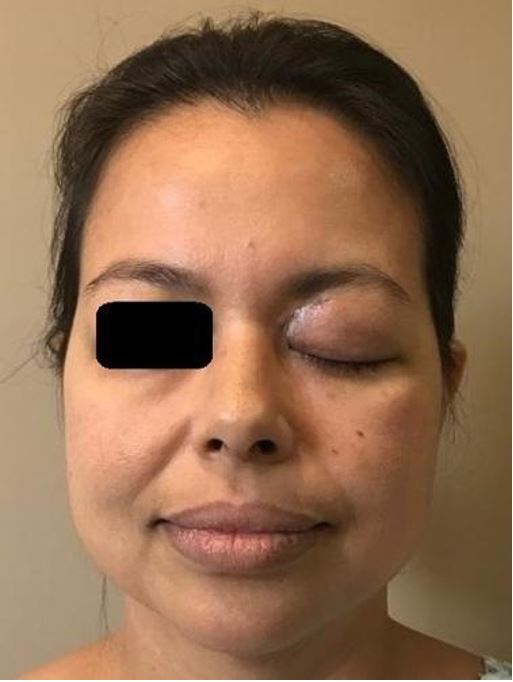
Involvement of the left orbit with edema, eyelid ptosis, purulent secretion and proptosis.

Three weeks after the sinusectomy, the patient was admitted to the emergency room of a tertiary care center with total paralysis on the left eye, fixed mydriasis, ptos, and amaurosis, compatible with orbital apex syndrome (Figure 1).

**Figure 2:**
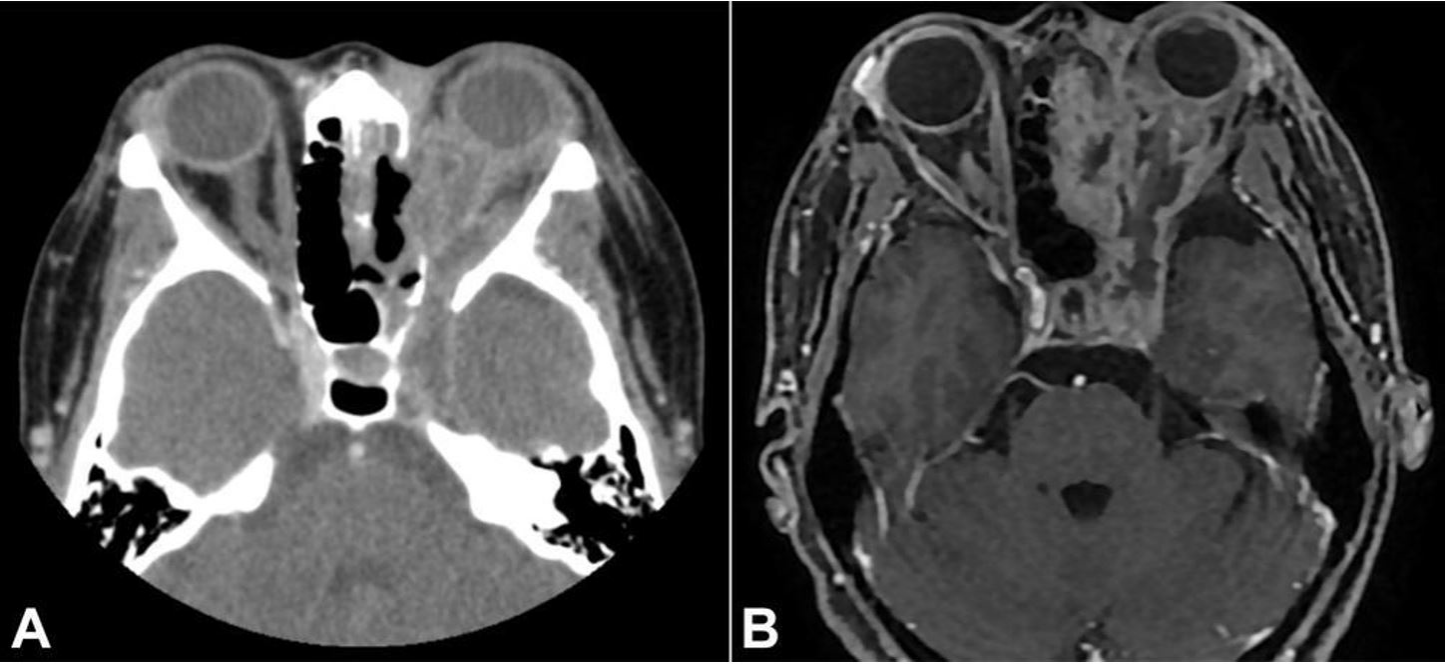
Computed tomography and magnetic resonance imaging of the orbital region: **A –** diffuse obliteration of the orbital fat on the left by tissue that involves the extraconal and intraconal compartments, more accentuated in the posterior portion of the orbit; **B –** diffuse thickening and peripheral enhancement of the sheath/optic nerve complex, suggesting direct infiltration by the fungal material.

Orbits and paranasal sinuses imaging exams showed findings consistent with invasive inflammatory disease, with orbital involvement on the left (Figure 2A, 2B).

**Figure 3:**
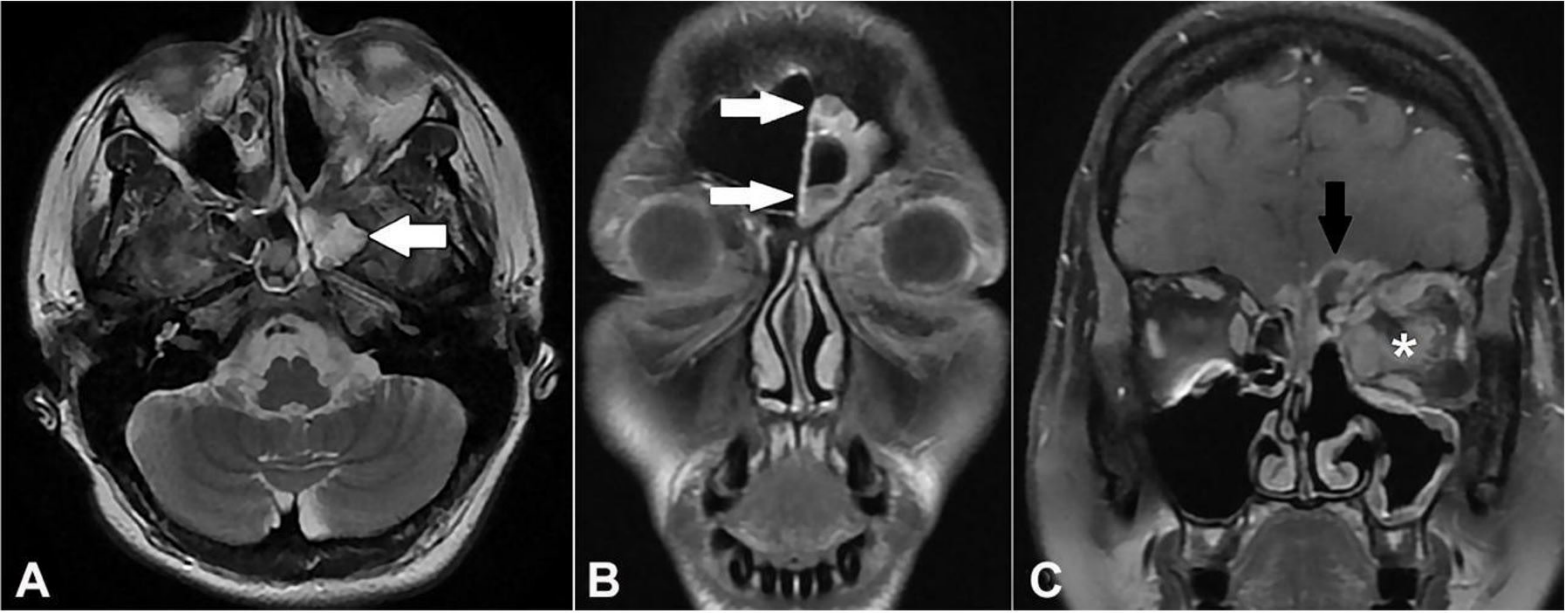
Resonance imaging of the orbital region. **A**, **B**, and **C –** signs of inflammatory sinusopathy on the left side (white arrows) associated on this side with heterogeneous infiltrative tissue orbitofacial (asterisk) extending to the skull base and intracranial (black arrow), with an image aspect compatible with the clinical diagnosis of a fungal process.

Four days after the last admission, she underwent a second sinusectomy. The material collected in the procedure confirmed the presence of hyphae, and morphological findings were consistent with *Mucor spp by* histological methods (Grocott histochemical method and Acid-Schiff histochemical method). Prior to this histopathological analysis, the patient had been treated with voriconazole (200 to 300 mg, 4 mg/kg, adjusted according to the serum level) for ten days, which was replaced by liposomal amphotericin at a dose of 7.5 mg/kg/day. After one week, Isavuconazole 200 mg/daily added was added because of lack of clinical improvement. The magnetic resonance imaging (MRI) showed intracranial involvement signs (Figure 3).

One month after the introduction of antifungal therapy, the control MRI showed an increase in frontal lobes involvement. A surgical approach was indicated for debridement. In a preoperative arterial occlusion test, left internal carotid thrombosis was diagnosed.

**Figure 4:**
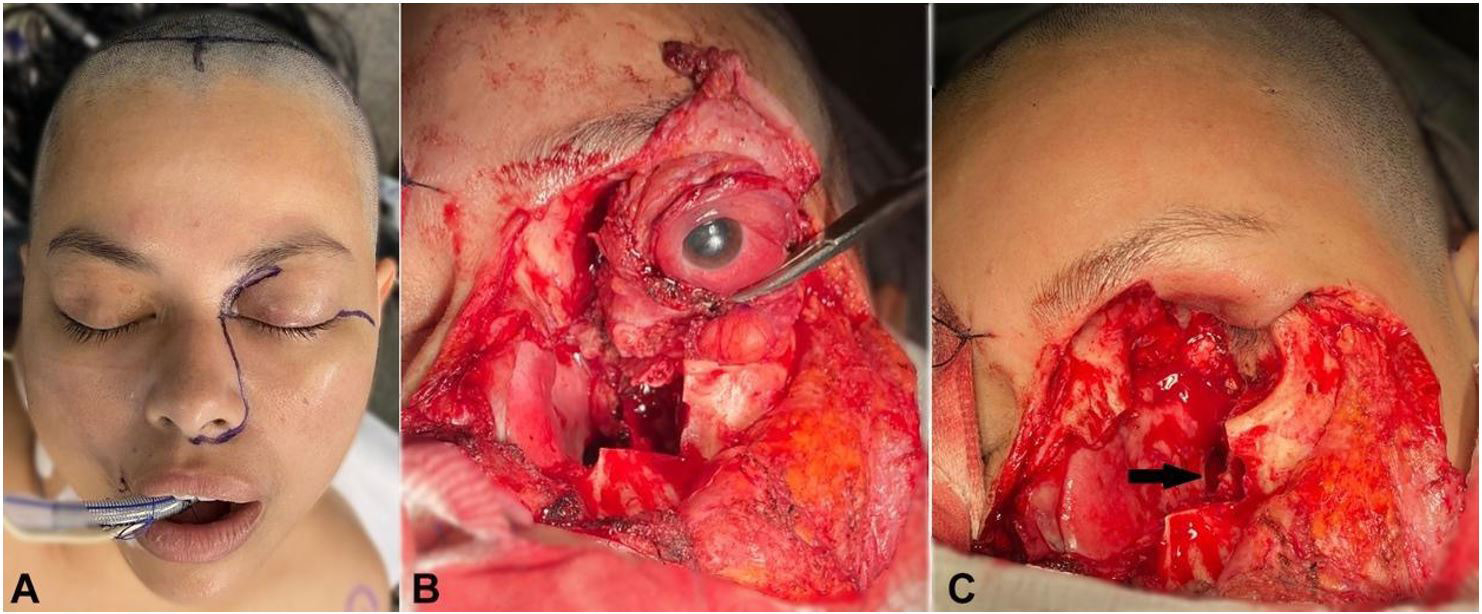
Surgical procedure: **A –** incision planning; **B –** maxillectomy of supra and mesostructures. Exposure of the left maxillary sinus. Orbital exenteration ongoing; **C –** after exenteration of the left orbit. Sphenoid sinusotomy (black arrow).

The Head and Neck Surgery and Neurosurgery teams undertook maxillectomy, ethmoidectomy, sphenoidectomy, orbit exenteration and exploration and debridement of the cavernous sinus on the left (Figure 4).

**Figure 5:**
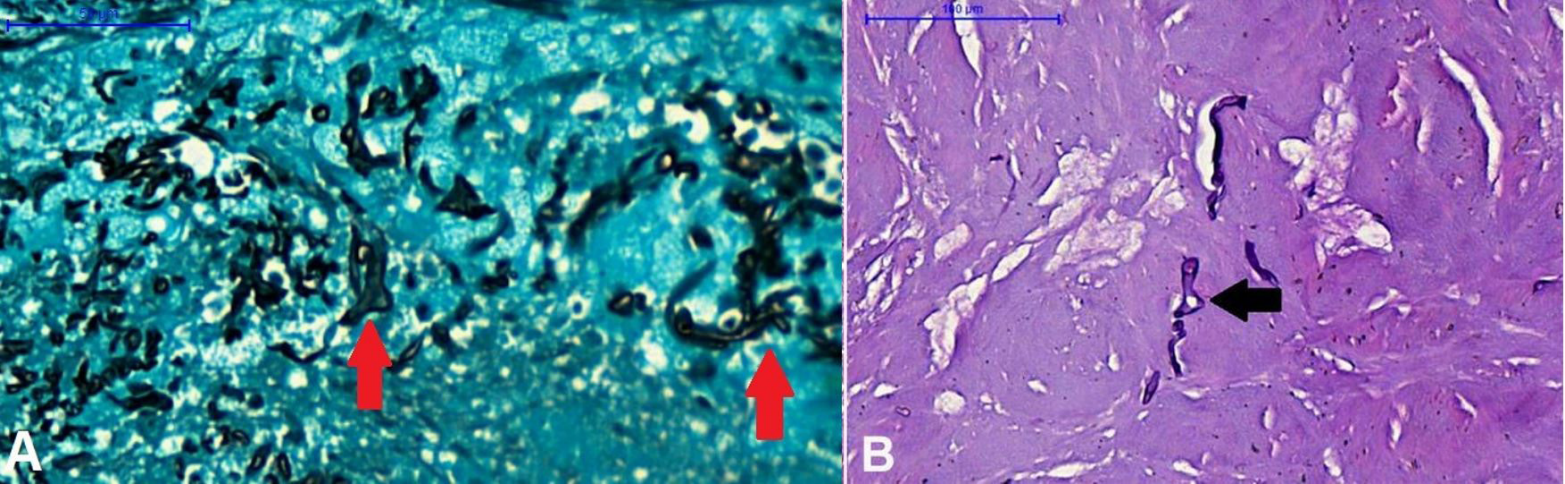
**A –** Grocott histochemical method, 400x magnification shows hyphae with diameters ranging from 1 to 5 μm and are hypo-septate (red arrows); **B –** Acid-Schiff histochemical method (PAS), 200x magnification. In some areas, the presence of irregular bifurcations (branching) that form 90-degree angles (black arrow) can be noted. **A-B** – The set of morphological findings suggests mucormycosis infection.

The histological examination confirmed the presence of mucor hyphae in the surgical specimen (Figure 5). During the postoperative period, she presented a cerebrospinal fluid fistula that was treated with acetazolamide and external lumbar cerebrospinal fluid drainage for 5 days.

**Figure 6:**
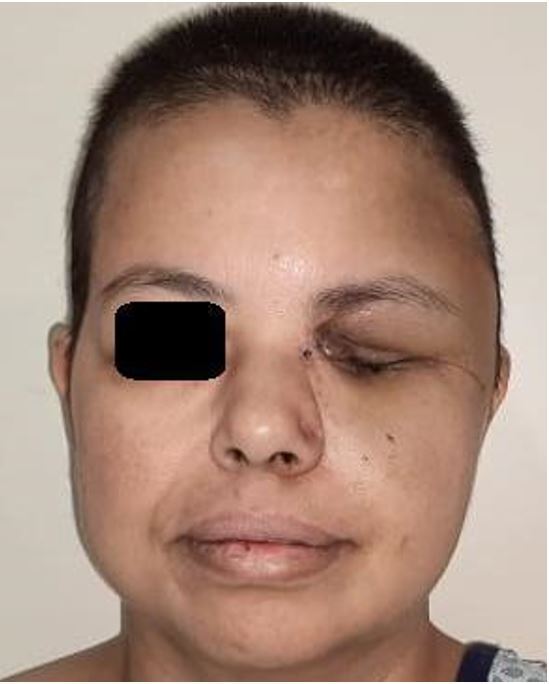
Patient’s face one month after the main surgery.

The patient was discharged from the intensive care unit on the twentieth postoperative day and had continued her treatment with liposomal amphotericin (achieving 9 mg/kg/day due to patient’s weight loss) for another six weeks and isavuconazole as long maintenance therapy. At the end of this report, the patient was in good general condition (Figure 6).

## DISCUSSION

COVID-19-associated mucormycosis has been reported worldwide, especially in India.^4^ In a review conducted by John et al.,^4^ 41 cases of patients with COVID-19-associated mucormycosis were identified. Among the studied cases, 94% involved diabetic patients. Eighty-eight percent of them received systemic corticosteroids, and 82.9% had a cranial presentation. Unlike most patients reported worldwide, our patient had no previous known immune compromise and was non-diabetic. However, like the other described cases, she received a low dose of corticosteroid treatment for COVID-19, and her clinical presentation was rhino-orbital-cerebral. In an Indian multicenter retrospective study composed of 187 COVID-19-associated mucormycosis cases, Patel et al.^3^ found that inappropriate glucocorticoid use (dexamethasone-equivalent doses >6 mg/day for >10 days) was independently associated with late COVID-19-associated mucormycosis (when mucormycosis diagnosis occurred ≥8 days after COVID-19 diagnosis).

Pasero et al.^5^ discussed whether Sars-Cov-2 itself could be a predisposing factor to immunosuppression and its consequent opportunistic infections. The tendency towards ketoacidosis, corticosteroid use, changes in iron metabolism and damage to the endothelium are possible COVID-19’s conditions that could be associated with mucormycosis.^4^ Except for dexamethasone therapy, no other evident predisposing factors were identified in the case described herein.

The patient reported here received optimized systemic antifungal therapy and extensive surgical treatment. So far, four months after the last hospital discharge, the patient has been in good general condition.

## CONCLUSION

We report the case of a non-diabetic, apparently previous immunocompetent patient with an extensive mucormycosis after COVID-19 infection treated with a short-term and low -dose of dexamethasone. More research is needed to understand the role of SARS-CoV-2 infection in the Mucormycosis’ development. Furthermore, it is important to emphasize the rational use of corticosteroids.
